# Identification of candidate chromosome region of *Sbwm1* for *Soil-borne wheat mosaic virus* resistance in wheat

**DOI:** 10.1038/s41598-020-64993-3

**Published:** 2020-05-15

**Authors:** Shubing Liu, Guihua Bai, Meng Lin, Mingcheng Luo, Dadong Zhang, Feng Jin, Bin Tian, Harold N. Trick, Liuling Yan

**Affiliations:** 1 State Key Laboratory of Crop Biology, College of Agronomy, Shandong Agricultural University, Taian, Shandong 271018 China; 2 USDA-ARS, Hard Winter Wheat Genetics Research Unit, 4008 Throckmorton Hall, Manhattan, KS 66506 USA; 3 Department of Agronomy, Kansas State University, Manhattan, KS 66506 USA; 4 Department of Plant Science, University of California- Davis, Davis, CA 95616 USA; 5 Chengdu Donnees Biotechnology Co., Ltd, Chengdu, Sichuan 610000 China; 6 Department of Plant Pathology, Kansas State University, Manhattan, KS 66506 USA; 7 Department of Plant and Soil Sciences, Oklahoma State University, Stillwater, OK 74078 USA

**Keywords:** Plant breeding, Plant breeding

## Abstract

*Soil-borne wheat mosaic virus* (SBWMV) causes a serious viral disease that can significantly reduce grain yield in winter wheat worldwide. Using resistant cultivars is the only feasible strategy to reduce the losses caused by SBWMV. To fine map the resistance gene *Sbwm1*, 205 wheat accessions was genotyped using wheat Infinium iSelect Beadchips with 90 K SNPs. Association analysis identified 35 SNPs in 12 wheat genes and one intergenic SNP in the *Sbwm1* region that showed a significant association with SBWMV resistance. Those SNPs were converted into Kompetitive Allele-Specific Polymerase assays (KASP) and analyzed in two F_6_-derived recombinant inbred line (RIL) populations derived from the crosses between two resistant cultivars ‘Wesley’ and ‘Deliver’ and a susceptible line ‘OK03825-5403-6’. Linkage analysis mapped this gene on chromosome 5D at intervals of 5.1 cM and 3.4 cM in the two populations, respectively. The two flanking markers in both populations delimited the gene to a 620 kb region where 19 genes were annotated. Comparative analysis identified a syntenic region of 660 kb in *Ae. tauschii* with 18 annotated genes and a syntenic region in chromosome 1 of *B. distachyon*. The candidate region includes several disease resistance related genes and we identified a PTI1-like tyrosine-protein kinase 1 gene as a putative candidate gene for *Sbwm1*. The two flanking SNPs for *Sbwm1* can effectively separate the resistant and susceptible lines in a new diversity panel of 159 wheat germplasm. The results from this study lay a solid foundation for the cloning, functional characterization and marker-assisted selection of *Sbwm1*.

## Introduction

Soil-borne wheat mosaic (SBWM) disease, caused by *Soil-borne wheat mosaic virus* (SBWMV) was first appeared in the Great Plains of the U.S.A. in 1919^1^ and has become an important disease in most winter wheat growing regions globally^2–7^. SBWMV infects wheat roots in wet soil during germination at 15 °C to 20 °C with an optimum temperature at 17 °C. Soil water is critical for the swimming zoospore to reach host roots^8,9^. Virus movement from the site of infection on roots to the leaves occurs at temperatures lower than 20 °C. Higher temperatures limit virus movement into leaves and further infection in developing leaves will not occur if temperatures rise to more than 20 °C after some leaves are infected^9^.

The SBWMV symptom usually first appears in early spring when the crop begins to green up. Low spring temperatures is usually conducive to the occurrence of SBWMV and could promote the development of the symptoms. Irregular chlorotic patches in a field indicate SBWMV infection. In dry environments, patches of symptomatic SBWMV-infected plants usually present in low-lying, wet regions of a field that are favorable for infection by the swimming zoospores of *Polymyxa graminis*, the vector of SBWMV^10^. In wet soil climates, these patches may present anywhere in an infected field. SBWMV symptoms stop developing on leaves when the average temperature exceeds 20 °C, but can be reinitiated on newly emerged leaves given conductive environmental conditions^10^. Reported yield losses caused by SBWMV ranged from 10% to 30% and can reach up to 80% in SBWMV severely occured fields in the U.S.A. and 50% in Brazil^11–13^.

*Polymyxa graminis* is one of the endoparasitic slime molds (Plasmodiophoromycota), which can produce resting spores that harbor viral RNA and movement protein, and can be easily distributed by wind, water and machinery. Those resting virus-containing spores can remain dormant and invasive in soil for up to 30 years, and then germinate in an environment favorable for infection when host are available. Thus, it is very difficult to control this disease once it happened in a field. The most practical SBWM control strategy is to grow resistant cultivars^2,14,15^.

Inheritance studies have shown that resistance to SBWMV is controlled by major genes. Winter wheat cultivars such as Shawnee, Centurk and KS73256 were reported to carry a dominant gene for SBWMV resistance. Newton might contain a gene for resistance different from that in ‘Arthur 71’, ‘Homestead’, and ‘Tascosa’^14^. However, a cultivar ‘Embrapa 16’ from Brazil was reported to carry two genes for SBWMV resistance^2^. With the rapid development and wide use of molecular markers in wheat genetic studies, quantitative trait locus (QTL) mapping approach has been used to localize SBWMV resistance genes using linkage maps. The QTL on 5DL has been reported by several independent studies on winter wheat ‘Karl 92’^16^, ‘Pioneer 26R61’, ‘AGS 2020’ and ‘Heyne’^12,17^. A breeding line KS96WGRC40 derived from *Aegilops tauschii* also carries this gene^18^. This gene was designated as *Sbwm1*^17^. Interestingly, a gene conferring resistance to *Soil-borne cereal mosaic virus* (SBCMV) in ‘Cadenza’, ‘Tremie’ and ‘Claire’, designated as *Sbm1*, was also mapped in the region of *Sbwm1*^19,20^. Because they tightly linked to the marker *Xgwm469-5D*, *Sbm1* for SBCMV resistance is either a tightly linked or the same gene as *Sbwm1* for SBWMV resistance.

Single nucleotide polymorphisms (SNPs) are the most widely distributed DNA sequence variations in a genome. During the past decade, next-generation sequencing (NGS) technologies have become the cheapest and fastest technology for genome-wide SNPs discovery^21^. Fixed SNP chips have been released and widely used for various genetic and breeding studies including genome-wide association analysis and genomic selection in many crop species^21–23^. In wheat, SNP chips with various number of SNPs including Wheat 9 K iSelect, Wheat 90 K iSelect and Wheat 660 K Axiom SNP chips have been developed and widely used to map genes or QTLs for different traits^24–28^.

In a former study, a SSR marker *Xgwm469*, that was associated with the SBWMV resistance on chromosome 5DL has been identified^29^. Further association mapping analysis using Wheat 9 K iSelect chip identified six SNPs from two genes that were highly associated with SBWMV resistance, and the identified SNPs were further mapped on 5DL using a F6 RIL population ‘Heyne’/‘Trego’^17^. However, those SNPs were mapped on one side of *Sbwm1* and the marker flanking on the other side of *Sbwm1* is about 20 cM a way. Therefore, a tightly linked flanking marker is urgently needed for effective marker-assisted transfer of *Sbwm1* to new wheat cultivars and cloning *Sbwm1*.

In this study, the panel of 205 wheat accessions previously genotyped with 9 K SNP chips were further genotyped using Infinium 90 K wheat SNP chips to identify more flanking SNPs for *Sbwm1*. Two new RIL populations were used to map the SBWMV resistance genes in the two resistant cultivars Wesley and Deliver and to verify the linkage among the newly identified SNP markers *Sbwm1*. We also pinpointed the candidate region for *Sbwm1* using the Chinese Spring reference genome, developed KASP assays for the linked SNPs to *Sbwm1* for marker-assisted selection (MAS), and identified a putative candidate gene for *Sbwm1* by gene expression analysis.

## Materials and Methods

### Plant materials

The association mapping panel includes 205 wheat accessions with 137 hard winter wheat (HWW) and 68 soft winter wheat (SWW) from six 2008 HWW and SWW nurseries (Supplementary Table S1). Seeds for DNA isolation and disease evaluation were originated from a single plant of each accession increased in a greenhouse to minimize within-line heterogeneity^29^. All these accessions were genotyped using Infinium iSelect 90 K wheat SNP chips^25^. A second panel of 159 wheat cultivars and breeding lines were used to validate the mapped KASP assays (Supplementary Table S2).

Two F_6_ RIL populations developed by single-seed descent from the crosses ‘Wesley’ x OK03825-5403-6 (180 RILs) and ‘Deliver’ x OK03825-5403-6 (260 RILs) were used to map the SBWMV resistance gene. ‘Wesley’ and ‘Deliver’ are SBWMV-resistant HWW cultivars, whereas OK03825-5403-6 is a highly SBWMV-susceptible HWW breeding line.

### Disease evaluation

The association panel of 205 wheat accessions were evaluated for SBWMV resistance in the SBWMV-infested field at the Rocky Ford Research Farm of Kansas State University, Manhattan, KS in 2009–2010 and 2010–2011 wheat growing seasons. The nursery has shown consistent and severe SBWMV infection on susceptible wheat cultivars since 2006. Field experimental design, planting, and disease damage evaluation followed Zhang *et al*.^30^. An additional panel of 159 accessions were evaluated for SBWMV resistance in 2010–2011 and 2011–2012 wheat growing seasons in the same field nursery for marker validation. The two RIL populations were evaluated for SBWMV resistance in the same field in 2013–2014 and 2014–2015 growing seasons using the same rating scale described for association mapping. All experiments used random complete block design with two replicates. In all experiments, each accession or RIL was sowed at 1.5 m -long rows with planting density of 40 seeds. After planting, the nursery was sprinkler irrigated everyday for 7 days till the seedling emergence to maintain high moisture in the soil to promote SBWMV infection.

### DNA extraction and 90 K SNP assay

The 205 accessions were genotyped using the 90 K SNP chips at USDA-ARS Cereal Crops Research Unit, Fargo, ND as described by Wang *et al*.^25^. SNPs with less than 5% minor allele frequency (MAF) or with more than 15% missing data were removed^31^. A total of 21,600 SNPs were scored and used in the final analysis. Sequences that harbored significant SNPs were blasted against the Chinese Spring Wheat SeqVer1.0 reference sequence^32^ to assign their putative chromosome positions and were converted to Kompetitive Allele Specific Polymerase Chain Reaction (KASP) assays for screening the two RIL populations (Table 1). KASP analysis following the methods described by Liu *et al*.^17^.

**Table 1:** Primers for KASP-SNPs that are highly associated with SBWMV resistance in the association mapping population.

SNP Name	Left Primer	Right Primers
BS00067308_51-FAM	ATGCTGCATCCACGTCCTt	CACGTCTTCACGGATGTTTTT
BS00067308_51-HEX	ATGCTGCATCCACGTCCTg	
CAP12_c5949_104-FAM	GCATCAGGAAGAGGAACAGCa	GCCGTCGTAGTAGGTGAAGG
CAP12_c5949_104-HEX	GCATCAGGAAGAGGAACAGCc	
BS00079676_51-FAM	GGGTACTCTCGTCTTCCTGCATa	CAGTACAAAGCGCAACCTCA
BS00079676_51-HEX	GGGTACTCTCGTCTTCCTGCATg	
Kukri_c5528_603-FAM	CCAGTTAAAGGCTACATGGAGAt	AGGCCACATGAATGAGATCC
Kukri_c5528_603-HEX	CCAGTTAAAGGCTACATGGAGAc	
BS00011469_51-FAM	TGACTGGAAGGAAACCGGTt	GCCCAAGTGACTAAACTTTGC
BS00011469_51-HEX	TGACTGGAAGGAAACCGGTc	
BS00073116_51-FAM	CCGTGTGATAAGGATTCTGGt	GTTGTGTGGAGCACAGCACT
BS00073116_51-HEX	CCGTGTGATAAGGATTCTGGg	
BS00105939_51-FAM	GGTTGAGCAGGAAGCAGGa	GCAAGCAGACCAGTCTCTCA
BS00105939_51-HEX	GGTTGAGCAGGAAGCAGGg	
Contig08110-FAM	CCACCAATGGACACAAGGc	TCATCGCTCCAGGAATTACA
Contig08110-HEX	CCACCAATGGACACAAGGt	
Excalibur_c22724_85-FAM	ACCATTCACAACAATTGCGt	GATAATTTGTGCCGCGAGTT
Excalibur_c22724_85-HEX	ACCATTCACAACAATTGCGc	
Excalibur_c2795_1518-FAM	ATATACAAGGTATATTAGGt	TAACCCACCACCTGGCTTCT
Excalibur_c2795_1518-HEX	ATATACAAGGTATATTAGGc	
BobWhite_c13030_406-FAM	ATGCTCCCGGGCTTGATa	CCCATCACGCTCGACTTC
BobWhite_c13030_406-HEX	ATGCTCCCGGGCTTGATg	

### Development of SNP markers using *Ae. tauschii* reference genome

To identify more markers in the SBWMV resistance QTL region, the probe sequences harboring the identified SNPs for SBWMV resistance were used to blast the draft *Ae. tauschii* reference genome^33^. Fourteen mapped SNPs (listed in Supplementary Table S4) in *Ae. tauschii* physical map in the QTL region were identified and converted to KASP assays to screen the three parents, Wesley, Deliver and OK03825-5403-6, of the two RIL populations, and the polymorphic SNP (Contig08110) was screened across the two RIL populations (Table 1).

### Statistical analysis

Data for SBWMV phenotypes were analyzed using two-way GLM in SAS for Windows v9 (SAS Institute, Cary, NC) to determine the effects of genotypes (g), environments (e), and G x E interactions. Heritability (H^2^) was estimated using the formula

$${{\rm{H}}}^{2}={6}_{{\rm{g}}}^{2}/({6}_{{\rm{g}}}^{2}+{6}_{{\rm{ge}}}^{2}/{\rm{n}}+{6}_{{\rm{e}}}^{2}/{\rm{nr}})$$

where б_g_^2^ is the variance among RILs, б_ge_^2^ is the variance for G x E; б_e_^2^ is the variance of environments, n is the number of environments, and r is the number of replicates^34^.

### Population structure, kinship, linkage disequilibrium and association analysis of the AM Panel

Population structure was assessed by Structure 2.3.4^35^ using a set of 1,500 SNPs that are evenly distributed on all the 21 wheat chromosomes^31^. The admixture model was used for structure analysis and the number of sub-populations (K) was set as 1 to 10 with variable length of burn-in period and number of iterations at 20,000–250,000. For each trait, Bayesian information criterion (BIC) was applied to determine the optimum number of subpopulations^31^. Kinship was calculated using the same set of 1,500 SNPs used for structure analysis and SPAGeDi package^36^.

Genome-wide association analysis used the generalized linear model (GLM) with the Q matrix as fixed effects and the mixed linear model (MLM) with a Q matrix as fixed effects and a kinship matrix as random effects. The models were applied to each experiment for SBWMV resistance, and model fitness was determined based on the Bayesian information criterion value.

Association analysis of SNP data was conducted using the genome association and prediction integrated tool (GAPIT) implemented in a R package^37^. A threshold of *p* < 10^−5^ (1/number of markers) was set to claim significant marker-trait associations. Linkage disequilibrium and haplotype analyses of the significant SNPs were performed using HAPLOVIEW v.4.2 (http://www.broadinstitute.org/scientific-community/science/programs/medical-and-population-genetics/haploview/haploview).

### Linkage mapping

Genotypes with disease rating of 1–2 were classified as resistant and 3–4 as susceptible for SBWMV resistance in the two RIL populations. SNP linkage maps were constructed for the two RIL populations using JoinMap ver. 4.0^38^. Recombination fractions were converted into centiMorgans (cM) using the Kosambi function^39^. The threshold of logarithm of odd (LOD) score was set at 3.0 to claim linkage among markers with 0.4 as a maximum fraction of recombination. The goodness-of-fit between observed and expected segregation ratios between two alleles was analyzed for each marker locus using a chi-square-test.

### Marker sequence analysis and candidate gene annotation

Sequences that contain significant SNPs associated with SBWMV resistance were used as queries to blast the IWGSC Chinese Spring reference genome sequence RefSeqv1.0, *Ae. tauschii* reference genome sequence and *B. distachyon* genome sequence using BLASTN^32,40,41^. A significant match was declared when the queried sequences showed at least 99% nucleotide identity with an e-value lower than e^−20^.

### RNA extraction and gene expression analysis

Leaf tissues were collected from seedlings of Wesley and OK03825-5403-6 grown in the SBWMV field nursery for RNA extraction in fall 2015 and spring 2016 when the SBWMV symptom appeared. RNA was extracted and purified using the RNeasy Plant Kit with on-column DNaseI treatment (Qiagen, Valencia, CA). Complementary DNA from reverse-transcription reaction using a SuperScript$R$II kit (Invitrogen, Grand Island, NY) was amplified by conventional reverse transcriptase-PCR (RT-PCR). The annotated genes in the target region were tested for the gene expression using semi-quantitative PCR analysis. Each PCR reaction mixture consisted of 15.5 μL H_2_O, 2.4 μL 10 × buffer, 2.4 μL 50 mM MgCl2, 1 μL of 10 mM dNTPs, 1 μL each primer at 20 μM, 2.5 U Taq polymerase, and 1 μL cDNA. Cycle conditions were 95 °C for 1 min, annealing at 55–60 °C for 1 min for different primers, and extension at 72 °C for 1 min. A final extension at 72 °C was performed for 10 min. Annealing temperatures and cycle conditions for each primer pair were optimized such that each PCR was in the linear range of amplification. PCR primers were listed in Supplementary Table S5.

The identified candidate gene *TraesCS5D01G531200* was further quantified with three replications by quantitative real-time PCR performed on an ABI7900HT instrument using SYBR Green (Thermo Fisher Scientific). Gene expression level was analyzed using the 2^−ΔΔCt^ method. Actin gene was used as the endogenous control with ACCTTCAGTTGCCCAGCAAT as the forward primer, CAGAGTCGAGCACAATACCAGTTG as the reverse primer.

## Results

### SBWMV resistance in the association and linkage mapping populations

**Figure 1:**
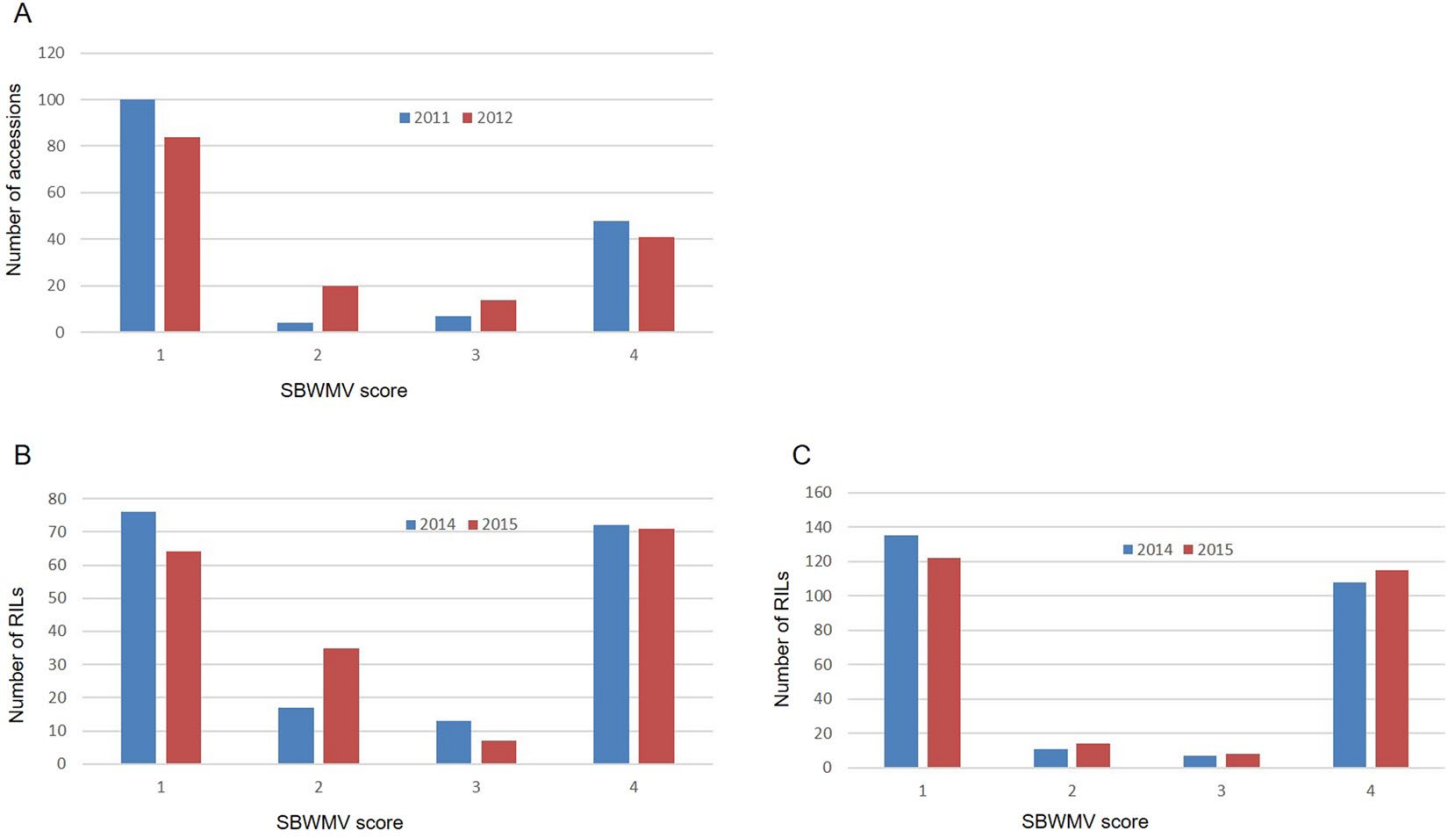
Frequency distribution of *Soil-borne wheat mosaic virus* disease scores in wheat accessions and recombinant inbred lines (RILs) evaluated in the field experiments at Manhattan, KS. (**A**) Accessions used for the KASP validation were phenotyped in the spring 2011 and 2012 field experiments; (**B**) RILs from Wesley x OK03825-5403-6 F_6_ population and (**C**) RILs from Deliver x OK03825-5403-6 population were phenotyped in the spring 2014 and 2015 field experiments. 1 = resistant, 2 = moderately resistant, 3 = moderately susceptible and 4 = susceptible.

High correlation coefficients were observed for SBWMV resistance between the two years of phenotypic data from the marker validation population (r = 0.92, *p* < 0.01), the two RIL populations (r = 0.81 and 0.94, *p* < 0.01), and the association mapping population (r = 0.85, *p* < 0.01)^17^, suggesting a high repeatability of SBWMV resistance data between the field experiments. Analysis of variance showed highly significant genotypic effects and very high heritabilities (from 84.9% to 98.2%) in both the RIL populations and the validation population (Supplementary Table S3). In the two biparental populations, the distribution of SBWMV disease scores deviated significantly from a normal distribution and showed an obvious bimodal distribution with two peaks towards the resistant and susceptible parents (Fig. 1), suggesting that a major gene is responsible for SBWMV resistance in Wesley and Deliver.

### Association analysis identified SNPs for the SBWMV resistance

**Figure 2:**
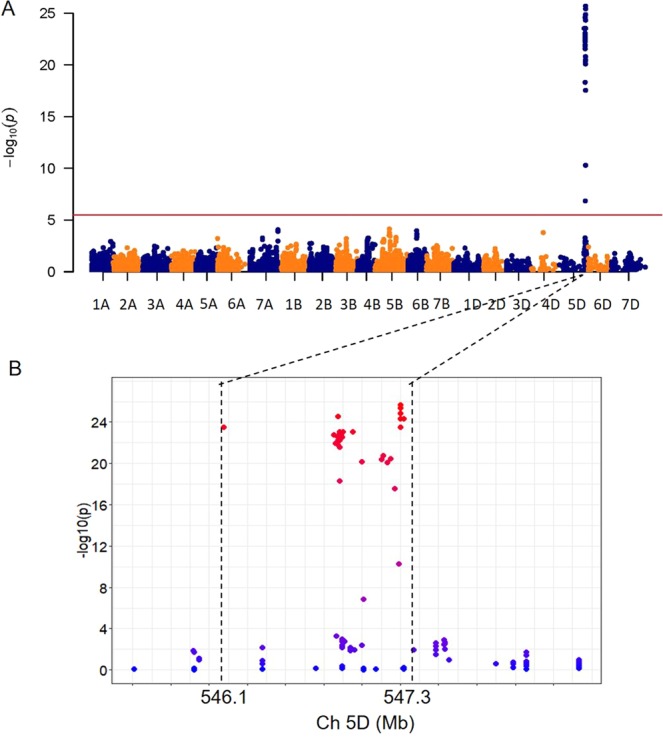
Manhattan plots for wheat resistance to *Soil-borne wheat mosaic virus* (SBWMV) in an association mapping population (**A**) and the physical positions of the significant SNPs on chromosome 5D (**B**). Red line represents the threshold to claim significant SNPs. Two-seasons of SBWMV ratings were collected from two field experiments plus the mean SBWMV ratings over both experiments.

Genotyping the association mapping population with the 90 K SNP chips identified 21,600 polymorphic SNPs. Using the MLM model, thirty-five SNPs were identified significantly associated with SBWMV resistance in both years (*P* < 10^−5^) (Fig. 2, Table 2), with 33 of them from 11 annotated high confidence genes (from *TraesCS5D01G529700* to *TraesCS5D01G532100*) on chromosome 5D. SNP *Excalibur_c22724_85* was from an annotated low confidence gene *TraesCS5D01G625100LC*, and BS00013935_51 was from an intergenic region. Using the Chinese Spring reference genome (IWGSC RefSeq v1.0), those SNPs can be located in a 69 Mb interval on 5D. However, when SNP *BS00013935_51*, the SNP with the lowest association among those SNPs (*P* = *1.44E-07*) was excluded, the interval can be delimited to a 1.18 Mb region from 546,086,597 to 547,273,657 bp on the chromosome 5D.

**Table 2:** Significant SNPs associated with *Soil-borne wheat mosaic virus* resistance and their corresponding annotated genes.

SNP Name	SNP Position	MAF^a^	Orthologous gene	FDR^b^
BS00013935_51	5D.480829545	0.35	Intergenic region	8.55E-5
BobWhite_c13030_406	5D.549670218	0.26	TraesCS5D01G529700	7.89E-21
CAP12_c5949_104	5D.550233279	0.31	TraesCS5D01G530200	2.16E-20
BS00079676_51	5D.550234521	0.33	TraesCS5D01G530300	1.05E-19
D_GA8KES401AL4GG_122	5D.550235948	0.31	TraesCS5D01G530300	2.93E-20
RAC875_c13169_459	5D.550235521	0.31	TraesCS5D01G530300	5.32E-20
BS00105939_51	5D.550235127	0.30	TraesCS5D01G530400	1.28E-19
BobWhite_c4438_162	5D.550272236	0.32	TraesCS5D01G530600	4.72E-20
BS00088587_51	5D.550272456	0.31	TraesCS5D01G530600	2.34-20
BS00088592_51	5D.550272362	0.37	TraesCS5D01G530600	3.15E-16
Excalibur_c687_886	5D.550271002	0.29	TraesCS5D01G530600	1.40E-21
Excalibur_c687_907	5D.550271023	0.31	TraesCS5D01G530600	2.00E-20
Excalibur_c687_961	5D.550271077	0.31	TraesCS5D01G530600	3.91E-20
IACX10520	5D.550272436	0.31	TraesCS5D01G530600	2.00E-20
Kukri_c1073_91	5D.550271228	0.31	TraesCS5D01G530600	1.74E-20
Kukri_c7786_81	5D.550241671	0.33	TraesCS5D01G530600	2.22E-19
wsnp_JD_c4438_5567834	5D.550272167	0.31	TraesCS5D01G530600	2.00E-20
wsnp_JD_c4438_5567972	5D.550272029	0.31	TraesCS5D01G530600	2.39E-20
wsnp_JD_c4438_5568170	5D.550271831	0.31	TraesCS5D01G530600	5.22E-20
D_GDS7LZN01CBWNE_99	5D.550282662	0.31	TraesCS5D01G530700	2.92E-20
Kukri_c5528_603	5D.550285051	0.31	TraesCS5D01G530700	1.74E-20
BS00073116_51	5D.550445864	0.35	TraesCS5D01G531100	4.36E-18
BS00011469_51	5D.550488971	0.34	TraesCS5D01G531200	1.24E-18
Excalibur_c14043_548	5D.550491920	0.38	TraesCS5D01G531200	1.82E-15
Excalibur_c28592_173	5D.550491839	0.36	TraesCS5D01G531200	2.35E-18
Excalibur_c28592_377	5D.550491410	0.36	TraesCS5D01G531200	5.53E-18
Excalibur_c42190_383	5D.550487798	0.36	TraesCS5D01G531200	2.96E-18
Excalibur_c2795_1518	5D.550503097	0.20	TraesCS5D01G531300	3.15E-08
BS00067308_51	5D.550837863	0.32	TraesCS5D01G531900	9.53E-22
BS00011794_51	5D.550854024	0.29	TraesCS5D01G532100	4.09E-22
BS00022036_51	5D.550854228	0.32	TraesCS5D01G532100	1.66E-21
CAP7_rep_c12715_390	5D.550853989	0.32	TraesCS5D01G532100	7.89E-21
CAP8_c145_89	5D.550893835	0.30	TraesCS5D01G532100	4.09E-22
wsnp_CAP11_c209_198671	5D.550854078	0.32	TraesCS5D01G532100	1.66E-21
Excalibur_c22724_85	5D.550362064	0.31	TraesCS5D01G625100LC	1.74E-20

^a^Minor allele frequency. ^b^adjusted P value.

Among the 11 high confidence genes that carry significantly associated SNPs for SBWMV resistance, *TraesCS5D01G530600* harbors 12 SNPs including nine new SNPs and three previously reported SNPs (*wsnp_JD_c4438_5567972, wsnp_JD_ c4438_5568170 and wsnp_JD_c4438_5567834*)^17^. *TraesCS5D01G532100* carries five SNPs with four new SNPs. All the remaining SNPs were distributed in nine other genes.

### Linkage mapping

To validate the marker-trait association, one SNP was selected from each of the 12 genes harboring the identified SNPs in the association mapping study for KASP assay conversion. The SNPs *wsnp_JD_c4438_5568170* from the gene *TraesCS5D01G530600* and *wsnp_CAP11_c209_198467* from the gene *TraesCS5D01G532100* (Table 1) were used to represent the two genes identified in the assays of 90 K wheat SNP chips. The 12 newly designed KASP-SNPs were used to screen the three parents, Deliver, Wesley, and OK03825-5403-6 for polymorphism, and ten of the newly designed KASP-SNPs were polymorphic between Wesley and OK03825-5403-6 and eight were polymorphic between Deliver and OK03825-5403-6, respectively. Those polymorphic SNPs also segregated in the two RIL populations.

**Figure 3:**
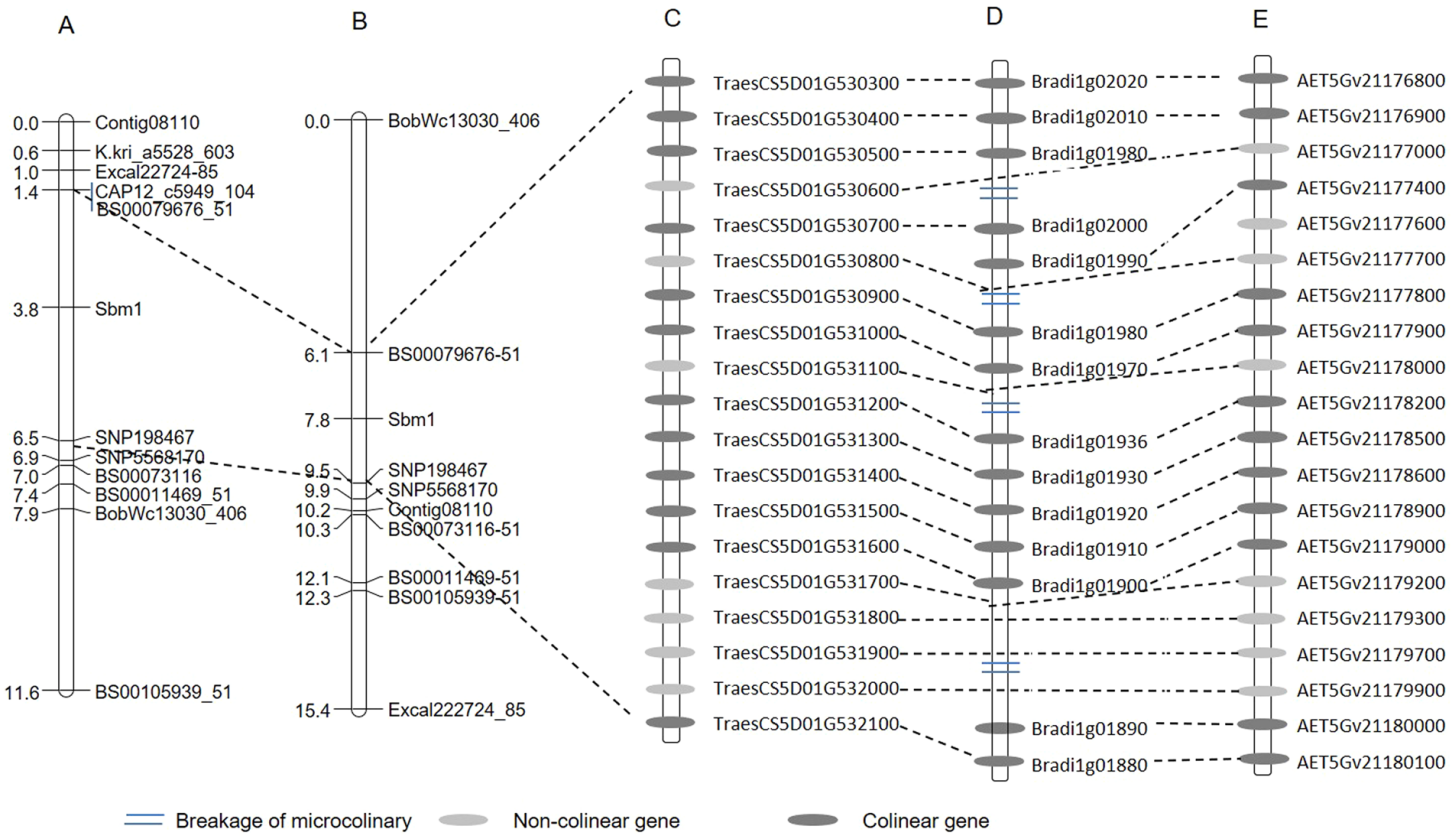
SNP maps of SBWMV-resistance gene (*Sbwm1*) on chromosome 5D and its syntenic regions in *B. distoschyn* and *Ae. tauschii*. (**A**) A SNP map of *Sbwm1* developed from the Wesley x OK03825-5403-6 F_6_ population; (**B**) A SNP map of *Sbwm1* developed from the Deliver x OK03825-5403-6 F_6_ population; (**C**) Annotated genes in the *Sbwm1* region flanked by the two closet markers based on the Chinese Spring reference genome; (**D**) The syntenic region in *B. distachyon* with homologous genes; and (**E**) The syntenic region of *Sbwm1* in *Ae. tauschii* with annotated genes.

BLASTN of the 35 marker sequences harboring the significant SNPs against *Ae. tauschii* physical map identified 14 SNP markers mapped in the physical map (Supplementary Table S4). Ten of the 14 SNPs were successfully converted into KASP assays, but only one SNP (Contig08110_553) showed polymorphism between the two pairs of parents and segregated in the two RIL populations. Together with previously designed 10 and 8 KASP-SNPs in the two populations, a total of 11 and 9 KASP-SNPs linked to SBWMV resistance were mapped on chromosome 5D and spanned 11.6 cM and 15.4 cM in Wesley x OK03825-5403-6 and Deliver x OK03825-5403-6 populations, respectively (Fig. 3). The two flanking markers, *wsnp_CAP11_c209_198467* and *BS0000079676_51* delimited the gene for SBWMV resistance to 5.1 cM and 3.4 cM intervals in the populations Wesley x OK03825-5403-6 and Deliver x OK03825-5403-6, respectively. The two flanking SNPs represent the two genes that are the closest to *Sbwm1*^17^ in the two populations, thus, the resistance gene in Wesley and Deliver was most likely the same as *Sbwm1* (Fig. 3A).

### Putative candidate genes in the genomic region of S*bwm1*

BLASTN of the sequences of the two flanking markers, *wsnp_CAP11_c209_198467* and *BS0000079676_51*, for *Sbwm1* against the Chinese Spring SeqVer1.0 reference genome identified a 620 kb interval in wheat on chromosome 5D (Fig. 3). Annotation of the genes in this interval revealed 19 high confidence genes and one low confidence gene (Fig. 3C, Supplementary Table S5). Comparative analysis between wheat and *Ae. tauschii*, the donor of D genome of hexaploid wheat, identified the conserved collinear orthologous region of *Sbwm1* with 20 high confidence orthologous genes including the two flanking genes (*AET5Gv21176900*–*AET5Gv21180000*) in a 660 kb interval on the chromosome 5D of *Ae. tauschii* (Supplementary Table S5). Among the 20 annotated genes of *Ae. tauschii*, 17 were the same to those in Chinese Spring wheat, and two genes (*AET5Gv21177600*, *AET5Gv21180000*) present in *Ae. tauschii*, but absent in wheat, and one gene (*TraesCS5D01G530500*) present in wheat, but absent in *Ae. tauschii* (Fig. 3E). Comparative analysis between wheat and *B. distachyon* identified a syntenic region (*Bradi1g01880* to *Bradi1g02020)* on chromosome 1. However, this synteny in *B. distachyon* was interrupted by four non-syntenic regions (Fig. 3D). Twelve genes were common between *B. distachyon* and wheat or *Ae. tauschii*.

Besides the two flanking genes, 17 genes were in the genomic interval annotated from *TraesCS5D01G530400* to *TraesCS5D01G532000*, which include genes encoding a sister chromatid cohesion protein DCC1 (*TraesCS5D01G530400*), a poly (ADP-ribose) glycohydrolase activity proteins *(TraesCS5D01G530500* and *TraesCS5D01G530900*), a protein kinase family protein (*TraesCS5D01G530600*), a potassium channel activity protein (*TraesCS5D01G530700*), a S-adenosylmethionine-dependent methyltransferase (*TraesCS5D01G530800*), a pentatricopeptide repeat domain family protein (*TraesCS5D01G531000*), a zinc ion binding protein and zinc finger containing proteins (*TraesCS5D01G531100* and *TraesCS5D01G531400*), a pto-interacting protein 1 (PTI1) (*TraesCS5D01G531200*), a protein with serine/threonine phosphatase activity (*TraesCS5D01G531300*), an albino-like protein (*TraesCS5D01G531600*), a neutral amino acid transmembrane transporter activity protein (*TraesCS5D01G531700*), a KDEL sequence binding protein (*TraesCS5D01G531800*), a major pollen allergen-like protein *(TraesCS5D01G531900*), and a gene encoding a protein with unknown function *(TraesCS5D01G531500*) (Supplementary Table S3).

**Figure 4:**
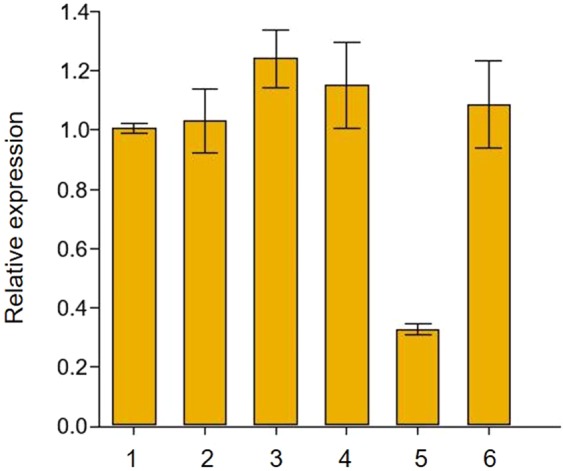
Gene expression analysis of *TraesCS5D01G531200* in leaf tissue in the resistant and susceptible parents. Leaf tissue were collected from Wesley (resistant) and OK03825-5403-6 (susceptible) in early spring from uninfected field (1, 2), before winter in infected field (3,4), and in early spring from infected field with SBWMV symptom present (5, 6). *Actin* was used as the endogenous control.

Expression test of these genes in leaves showed that eight genes did not express (*TraesCS5D01G530400, TraesCS5D01G530400, TraesCS5D01G531400, TraesCS5D01G531500, TraesCS5D01G531700, TraesCS5D01G531900, TraesCS5D01G531200, TraesCS5D01G625100LC*), whereas nine expressed but showed the same expression levels between the resistant and susceptible parents (*TraesCS5D01G530600, TraesCS5D01G530700, TraesCS5D01G530800, TraesCS5D01G530900, TraesCS5D01G531000, TraesCS5D01G531100, TraesCS5D01G531300, TraesCS5D01G531600, TraesCS5D01G531800*) before and after SBWMV symptoms appeared. Only one gene, *TraesCS5D01G531200* showed significant differential expression between the resistant and susceptible parents when SBWMV symptoms appeared in the leaf tissues (Fig. 4), indicating that SBWMV infection differentially induced expression of *TraesCS5D01G531200*. Considering that *TraesCS5D01G531200* was the only one of the three genes (the other two were *TraesCS5D01G530600* and *TraesCS5D01G531300*) in this region encoding proteins that may be involved in disease resistance, we believed *TraesCS5D01G531200* might play an important role in the SBWMV resistance.

### Validation of the flanking markers in cultivars and breeding lines

To further validate the utility of markers that are tightly linked to *Sbwm1* in wheat breeding, the two flanking KASP markers, *wsnp_CAP11_c209_198467* and *BS0000079676_51*, were used to screen 159 additional wheat cultivars and breeding lines from U.S. hard winter wheat breeding programs and the validation panel was also phenotyped for SBWMV resistance in the field experiments (Table 3, Supplementary Table S2). SBWMV resistance of those accessions was consistent between two years with a high correlation of 0.91 (*p* < *0.001*). Among these accessions, 104 were resistant and 55 were susceptible. The two SNPs identified four haplotypes across these accessions (Table 3) with the A-A haplotype associated with resistant accessions, and G-G haplotypes with susceptible accessions. For SNP *wsnp_CAP11_c209_198467*, A presents in 99% of the resistant accessions and G presents in 98.2% of susceptible accessions. For SNP *BS00079676_51*, A and G alleles present in 95.2 and 96.6% of the resistant and susceptible accessions, respectively. Two recombinant haplotypes A-G and G-A were identified in 5 and 3 accessions, respectively. All the accessions with the A-G haplotype were resistant. For G-A haplotype, however, only one G-A accession was resistant and other two accessions were susceptible, indicating there is recombination between *Sbwm1* and *BS00079676_51* in these two accessions. The two KASP assays together can effectively distinguish resistance and susceptibility alleles in the diverse wheat accessions and can be used for the selection of *Sbwm1* in breeding programs.

**Table 3:** Haplotypes and SBWMV reactions of the two flanking SNPs for *Sbwm1* in 159 wheat cultivars and breeding lines.

*wsnp_CAP11_ c209_198467*	*B00079676_51*	Accession num	SBWMV resistance
A	A	99	resistant
A	G	5	resistant
G	G	52	susceptible
G	A	4	resistant (1), susceptible (3)

## Discussion

Severity of SBWM infection is affected by environmental factors such as soil water content after planting and temperatures in early spring. In this study, highly repeatable phenotypic data was generated from the two RIL populations, the association mapping population and a validation panel of the 159 wheat accessions in repeated experiments by carefully managing the SBWMV nursery. In the two RIL populations, a slight difference in disease ratings between two years was observed for some RILs (Fig. 1). The rating changes usually occurred within the resistant or susceptible categories, not between the two categories, which can be visualized by high correlation coefficients (from 0.81 to 0.94) of SBWMV resistance between years for the four populations investigated. Those results indicated that SBWMV resistance evaluated in the field nursery in this study was highly repeatable, thus is adequate for QTL analysis.

Our previous association mapping study identified an SSR marker, *Xgwm469* and two SNPs *wsnp_JD_c4438_5568170*, *wsnp_CAP11_c209_198467* from 9 K SNP chips that closely linked with *Sbwm1*^17,30^. In the current study, we used the new 90 K wheat SNP chips with high marker density to conduct a genome-wide association study on the same association mapping panel. We found 21,600 polymorphic SNPs from the 90 K SNP chips, which is three times of those identified from the 9 K chips (6,895 SNPs). Among the polymorphic SNPs, 35 SNPs were identified to be highly associated with *Sbwm1*. More trait associated SNPs identified in this study is mainly due to the high SNP density (10 times) in the 90 K SNP chip than the 9 K SNP chip^25^. Among those *Sbwm1*-associated SNPs, *wsnp_JD_c4438_5568170*, *c4438_5567972*, *wsnp_JD_c4438_5567834* and *wsnp_CAP11_c209_198671* were the same as previously identified from the 9 K SNP chips, and the other 31 were newly identified SNPs from the new 90 K chips in this study, indicating that increasing marker density facilitate identification of more trait-associated SNPs. Those common SNPs identified in both chips were owing to that the most probes in the 9 K chip were included in the 90 K chip^25^.

Several independent mapping studies have identified a major locus for SBWMV resistance in the similar location on the long arm of chromosome 5D from different wheat cultivars and an *Ae. tauschii*-derived breeding line^12,16,18^. Those QTLs most likely underline the same gene, *Sbwm1* as reported by Liu *et al*.^17^. A gene for SBCMV resistance, designated as *Sbm1*^19^, was also mapped in the similar location as *Sbwm1* in wheat and they may be either tightly linked or the same gene.

In the linkage maps from Wesley/OK03825-5403-6 and Deliver/OK03825-5403-6 populations, those significant SNPs identified from the AM panel were all mapped near the SBWMV resistance gene in the same linkage group corresponding to the distal end of chromosome 5DL (Fig. 3). The SBWMV resistance gene was flanked by *BS00079676_51* and *wsnp_CAP11_c209_198467* with *BS00079676_51* as the closest marker in the two populations, respectively. Since *wsnp_CAP11_c209_198467* has been identified as the closest marker to *Sbwm1* in cultivar Heyne^17^, the resistance gene in Wesley and Deliver is in the same region of *Sbwm1* and they are most likely the same gene. Therefore, this study defined *Sbwm1* region to a 3.4 cM region in the Deliver/OK03825-5403-6 population and identified closely linked flanking markers for *Sbwm1*, which laid solid ground for map-based cloning and marker-assisted breeding of *Sbwm1*. Using association mapping together with linkage mapping can effectively determine the marker-trait association.

Using the IWGSC Chinese Spring reference genome RefSeqv1.0, all the newly identified SNPs were mapped in the interval between 546,086,597 and 547,273,657 on 5D. However, the two flanking SNPs further delimited the 3.4 cM *Sbwm1* region to a physical region of 620 kb (Fig. 3), suggesting *Sbwm1* is in a high recombination region. In this region, only 17 high confidence genes were annotated in wheat RefSeqv1.0 reference. Using *Ae. tauschii* reference genome, *Sbwm1* was delimited to a 660 kb region on 5D with 18 annotated genes (Fig. 3C,E). Only one predicted wheat gene, is absent in *Ae. tauschii*, and two *Ae. tauschii* genes are absent in wheat (Supplementary Table S5), indicating good microcolinearity between the two species in *Sbmv1* region. In the *Sbwm1* genomic region, we identified three candidate genes that are involved in disease resistance. Gene expression analysis identified only one gene showed significantly differential expression between the resistant parents and the susceptible parent (Fig. 4) in the tissues with SBWMV symptoms, which is selected as the candidate for further function analysis.

Wheat SNP chip may not be cost effective for breeding selection due to high cost per sample if only a few SNPs are interested. KASP assay is a time saving and cost-effective genotyping assay for single SNP analysis and has been successfully applied in wheat research and breeding^42–44^. In this study, all the identified SNPs that were highly associated with *Sbwm1* were converted into KASP assays to map *Sbwm1* in the two RIL populations. The KASP assays for those SNPs developed in this study will facilitate isolation and marker-assisted selection of *Sbwm1* in wheat.

The effectiveness of the two flanking KASP assays *wsnp_CAP11_c209_198467* and *BS00079676_51* were also tested in another panel of 159 wheat cultivars and breeding lines. The result showed that when an accession carries the two SNPs in coupling phase, the accession consistently showed expected (resistant or susceptible) phenotypes. However, six accessions carry the two SNPs in repulsion phase and inconsistent phenotypes were found for each recombinant haplotype. When allele A (resistance allele) at *wsnp_CAP11_c209_198467* and allele G (susceptibility allele) at *BS00079676_51* were together, all accessions with this haplotype showed SBWMV resistance; whereas when accessions carry G (susceptibility allele) at *wsnp_CAP11_c209_198467* and A at *BS00079676_51* (resistance allele), their reactions to SBWMV infection were mixed (Table 3), indicating that *Sbwm1* located between the two SNPs, and the two SNP together can separate the resistant and susceptible genotypes effectively and can be widely used in marker-assisted breeding to improve SBWMV resistance.

## Supplementary information

Supplementary information
