# Supplementary material for: Identification of candidate chromosome region of Sbwm1 for Soil-borne wheat mosaic virus resistance in wheat
Source: Sci Rep. 2020 May 15;10:8119. doi: 10.1038/s41598-020-64993-3 (PMC7229111; doi:10.1038/s41598-020-64993-3)
Supplement: Supplementary file 1 — Supplementary information [file 41598_2020_64993_MOESM1_ESM.doc]

**Identification of candidate chromosome region of *Sbwm1* for *Soil-borne wheat mosaic virus* resistance in wheat**

Shubing Liu^1*^, Guihua Bai^2,3*^, Meng Lin^3^, Mingcheng Luo^4^, Dadong Zhang^3^, Feng Jin^3,5^, Bin Tian^6^, Harold N Trick^6^, Liuling Yan^7^

^1^ State Key Laboratory of Crop Biology, College of Agronomy, Shandong Agricultural University, Taian, Shandong 271018, China.

^2^ USDA-ARS, Hard Winter Wheat Genetics Research Unit, 4008 Throckmorton Hall, Manhattan, KS 66506, USA.

^3^ Department of Agronomy, Kansas State University, Manhattan, KS 66506, USA.

^4^ Department of Plant Science, University of California- Davis, Davis, CA 95616, USA.

^5^ Chendu Donnees Biotechnology Co., Ltd, Chengdu, Sichuan 610000, China.

^6^Department of Plant Pathology, Kansas State University, Manhattan, KS 66506, USA.

^7^Department of Plant and Soil Sciences, Oklahoma State University, Stillwater, OK 74078, USA.

Correspondence Email: sbliu@sdau.edu.cn and guihua.bai@ars.usda.gov

Supplementary Information

Supplementary Tables

Supplementary Table S1 SBWMV resistance of accessions used for GWAS

Supplementary Table S2 Wheat cultivars and breeding lines used to validate the KASP assays for *Soil-borne wheat mosaic virus* resistance

Supplementary Table S3 Analysis of variance for SBWMV resistance in the RIL populations derived from crosses Deliver/OK03825-5403-6, Wesley/ OK03825-5403-6 and a natural population based on experiments conducted in Kansas State University, Manhattan KS, USA

Supplementary Table S4 Identified SNP marker in *Sbwm1* region on *Ae. tauschii* physical map

Supplementary Table S5 Annotation of genes in the *Sbwm1* region

Supplementary Tables

Supplementary Table S1 SBWMV resistance of accessions used for GWAS

| **Accession Name** | **Class** | **SBWMV resistance** ^a^ |
| --- | --- | --- |
| AGS 2000 | SWW | S |
| Antelope | HWW | R |
| AP04T8211 | HWW | R |
| AP05T2413 | HWW | S |
| AP06T3832 | HWW | R |
| AR96077-7-2 | SWW | R |
| AR97044-10-2 | SWW | R |
| AR97124-4-3 | SWW | R |
| Arena exp. | SWW | R |
| Atlas66 | SWW | S |
| B030543 | SWW | S |
| Bess | SWW | R |
| Branson | SWW | R |
| Centerfield | HWW | MR |
| Century | HWW | S |
| Chisholm | HWW | R |
| Clark | SWW | R |
| CO02W237 | HWW | R |
| CO03064 | HWW | S |
| CO03W043 | HWW | R |
| CO03W054 | HWW | S |
| CO03W139 | HWW | R |
| CO03W239 | HWW | S |
| CO04W210 | HWW | R |
| Coker 9553 | SWW | R |
| D04*5513 | SWW | S |
| D04-5012 | SWW | S |
| Deliver | HWW | R |
| Duster | HWW | R |
| Endurance | HWW | MR |
| Ernie | SWW | S |
| Freedom | SWW | MS |
| Fuller | HWW | R |
| G41732 | SWW | S |
| G59160 | SWW | R |
| G61505 | SWW | R |
| G69202 | SWW | S |
| GA991209-6E33 | SWW | S |
| GA991227-6A33 | SWW | R |
| GA991336-6E9 | SWW | S |
| GA991371-6E13 | SWW | S |
| Guymon | HWW | R |
| HV9W02-942R | HWW | R |
| HV9W03-539R | HWW | S |
| HV9W03-696R-1 | HWW | R |
| HV9W05-881R | HWW | R |
| HV9W96-1271R-1 | HWW | R |
| IL00-8530 | SWW | R |
| IL02-18228 | SWW | R |
| IL02-19463 | SWW | R |
| India exp. | SWW | R |
| INW0411 | SWW | R |
| Jerry | HWW | S |
| KS010143K-11 | HWW | R |
| KS010379M-2 | HWW | R |
| KS010514-9TM-10 | HWW | R |
| KS010957K~4 | HWW | R |
| KS020304K~3 | HWW | R |
| KS05HW121-2 | HWW | R |
| KS05HW136-3 | HWW | R |
| KS05HW15-2 | HWW | R |
| KS07HW117 | HWW | S |
| KS07HW25 | HWW | S |
| KS07HW81 | HWW | R |
| KS970093-8-9-#1 | HWW | R |
| KS970187-1-10 | HWW | R |
| KS980512-2-2 | HWW | R |
| KS980554-12-~9 | HWW | R |
| KY96C-0769-7-3 | SWW | R |
| KY97C-0321-02-01 | SWW | R |
| KY97C-0519-04-07 | SWW | R |
| LA01*425 | SWW | R |
| LA01138D-52 | SWW | S |
| LA02-923 | SWW | R |
| LA98214D-14-1-2-B | SWW | R |
| LA99005UC-31-3-C | SWW | S |
| M03-3616-C | SWW | MR |
| M04*5109 | SWW | R |
| M04-4566 | SWW | MR |
| M04-4715 | SWW | R |
| M04-4802 | SWW | R |
| MD01W233-06-1 | SWW | R |
| MD99W483-06-9 | SWW | MR |
| MO011126 | SWW | R |
| MO040152 | SWW | R |
| MO040192 | SWW | R |
| Mocha exp. | SWW | R |
| MT0495 | HWW | S |
| MTS04120 | HWW | S |
| MTS0531 | HWW | S |
| N02Y5117 | HWW | S |
| N98L20040-44 | HWW | S |
| NC03-6228 | SWW | R |
| NC04-15533 | SWW | MR |
| NE02533 | HWW | R |
| NE02558 | HWW | MR |
| NE04424 | HWW | R |
| NE04490 | HWW | R |
| NE05426 | HWW | R |
| NE05430 | HWW | R |
| NE05496 | HWW | R |
| NE05548 | HWW | R |
| NE05549 | HWW | R |
| NE05569 | HWW | R |
| NE06436 | HWW | R |
| NE06472 | HWW | S |
| NE06549 | HWW | S |
| NE06619 | HWW | S |
| NI04420 | HWW | R |
| NI04427 | HWW | R |
| NW03666 | HWW | S |
| NW04Y2188 | HWW | MS |
| NW05M6011-6-1 | HWW | S |
| NW05M6015-25-4 | HWW | R |
| NX03Y2489 | HWW | S |
| NX04Y2107 | HWW | S |
| OH02-12678 | SWW | R |
| OH02-7217 | SWW | S |
| OH03-41-45 | SWW | R |
| OK Bullet | HWW | R |
| OK00514-05806 | HWW | R |
| OK01420W | HWW | R |
| OK02405 | HWW | R |
| OK02522W | HWW | R |
| OK03305 | HWW | R |
| OK03522 | HWW | R |
| OK03716W | HWW | R |
| OK03825-5403-6 | HWW | S |
| OK04505 | HWW | R |
| OK04507 | HWW | R |
| OK04525 | HWW | R |
| OK05122 | HWW | R |
| OK05128 | HWW | R |
| OK05134 | HWW | R |
| OK05212 | HWW | R |
| OK05312 | HWW | R |
| OK05511 | HWW | R |
| OK05723W | HWW | MR |
| OK05830 | HWW | R |
| OK05903C | HWW | R |
| OK06210 | HWW | R |
| OK06313 | HWW | R |
| OK06319 | HWW | R |
| OK06336 | HWW | R |
| OK06345 | HWW | R |
| OK06518 | HWW | R |
| OK06528 | HWW | R |
| OK06848W | HWW | R |
| Overley | HWW | R |
| P02444A1-23-9 | SWW | S |
| P03112A1-7-14 | SWW | R |
| P03207A1-7 | SWW | R |
| P04287A1-10 | SWW | R |
| Pioneer Brand 26R61 | SWW | R |
| Roane | SWW | R |
| Scout 66 | HWW | S |
| SD03164-1 | HWW | R |
| SD05118 | HWW | S |
| SD05210 | HWW | R |
| SD05W030 | HWW | R |
| SD05W148-1 | HWW | S |
| SD06069 | HWW | S |
| SD06165 | HWW | R |
| SD06173 | HWW | S |
| SD06W117 | HWW | R |
| SD07204 | HWW | S |
| SD07220 | HWW | S |
| SD07W041 | HWW | R |
| T151 | HWW^a^ | R |
| T153 | HWW | R |
| T154 | HWW | R |
| T158 | HWW | R |
| TAM-107 | HWW | S |
| TAM-110 | HWW | R |
| TN801 | SWW | S |
| Trego | HWW | R |
| TX01V5134RC-3 | HWW | MS |
| TX02A0252 | HWW | S |
| TX03A0148 | HWW | S |
| TX03A0563 | HWW | R |
| TX04A001246 | HWW | R |
| TX04M410164 | HWW | S |
| TX04M410211 | HWW | R |
| TX04V075080 | HWW | R |
| TX05A001334 | HWW | R |
| TX05V5614 | HWW | S |
| TX06A001084 | HWW | S |
| TX06A001239 | HWW | S |
| TX06A001376 | HWW | S |
| TX06A001431 | HWW | S |
| TXHT001F8-CS06/325-PRE07/75 | HWW | MS |
| TXHT005F8-CS06/540-STA07/14 | HWW | -- |
| TXHT006F8-CS06/472-STA34 | HWW | MS |
| TXHT023F7-CS06/607-STA07/40 | HWW | S |
| U07-698-9 | HWW | R |
| USG 3555 | SWW | R |
| VA03W-412 | SWW | MR |
| VA04W-259 | SWW | R |
| VA05W-258 | SWW | R |
| VA05W-414 | SWW | MS |
| VA05W-78 | SWW | S |
| W06-202B | SWW | S |
| W98007V1 | SWW | R |
| W98008J1 | SWW | R |
| Wesley | HWW | R |
| a, R-resistent, MR-moderate resistant, MS-moderate susceptible, S-susceptible, --, missing | | |

Supplementary Table S2 Wheat cultivars and breeding lines used to validate the KASP assays for *Soil-borne wheat mosaic virus* resistance

| Accession_name | wsnp_CAP11_c209_198467 | BS00079676_51 | Appendix |
| --- | --- | --- | --- |
| OK07231 | TT | AA | cultivar |
| Jagger | TT | AA | cultivar |
| NW07534 | TT | AA | cultivar |
| NE06469 | CC | GG | cultivar |
| NE06607 | CC | GG | cultivar |
| NE07521 | CC | GG | cultivar |
| NE07531 | CC | GG | cultivar |
| NE07627 | TT | AA | cultivar |
| BC01007-7 | TT | AA | breeding line |
| 00X0100-51 | TT | AA | breeding line |
| HV9W04-1594R | TT | AA | breeding line |
| HV9W05-1125R | TT | GG | breeding line |
| CA9W06-788 | CC | GG | breeding line |
| CA9W07-817 | CC | GG | breeding line |
| CA9W08-856 | CC | GG | breeding line |
| BZ9W05-2039 | CC | GG | breeding line |
| BZ9W05-2043 | CC | GG | breeding line |
| MTS0532 | CC | GG | cultivar |
| MTS0713 | TT | AA | cultivar |
| TAM-107 | CC | GG | cultivar |
| Everest | TT | AA | cultivar |
| KS07HW52-5 | CC | GG | cultivar |
| KS08HW176-4 | CC | GG | cultivar |
| OK05526 | TT | AA | cultivar |
| OK02405 | TT | AA | cultivar |
| OK05204 | TT | AA | cultivar |
| T166 | TT | GG | cultivar |
| T167 | TT | AA | cultivar |
| T168 | TT | AA | cultivar |
| NE06545 | TT | AA | cultivar |
| NE07444 | TT | AA | cultivar |
| NI07703 | TT | AA | cultivar |
| NI08708 | TT | AA | cultivar |
| BC01131-24 | TT | AA | breeding line |
| BC01139-1 | TT | AA | breeding line |
| HV9W06-1046 | TT | AA | breeding line |
| HV9W06-509 | TT | AA | breeding line |
| HV9W06-262 | TT | AA | breeding line |
| CO04393 | CC | GG | cultivar |
| CO04499 | CC | GG | cultivar |
| CO050270 | CC | AA | cultivar |
| CO050303-2 | CC | GG | breeding line |
| KS010990M~8 | TT | AA | breeding line |
| KS06O3A~50-3 | TT | AA | breeding line |
| KS06O3A~58-2 | TT | AA | breeding line |
| KS011327M~2 | TT | AA | breeding line |
| OK07209 | TT | AA | breeding line |
| TX05A001822 | TT | AA | breeding line |
| TX06A001263 | CC | GG | breeding line |
| TX06A001132 | CC | GG | breeding line |
| TX05V7259 | CC | GG | breeding line |
| TX05V7269 | CC | GG | breeding line |
| TX05A001188 | CC | GG | breeding line |
| BC01138-5 | CC | GG | breeding line |
| AP06T3621 | TT | AA | breeding line |
| KS020648-M-6 | TT | AA | breeding line |
| KS020822-M-5 | TT | AA | breeding line |
| KS020947-K-13 | TT | AA | breeding line |
| KS021006-NT-9 | TT | AA | breeding line |
| KS030024-K-3 | TT | AA | breeding line |
| KS030024-K-4 | TT | AA | breeding line |
| KS030049-NT-7 | TT | AA | breeding line |
| KS030101-M-2 | TT | AA | breeding line |
| KS030124-K-4 | TT | AA | breeding line |
| KS031027-FHB~8 | TT | AA | breeding line |
| KS07F5BULK01-K-7 | TT | AA | breeding line |
| KS08FHB-31 | TT | AA | breeding line |
| KS08FHB-78 | TT | AA | breeding line |
| KS08IFAFS1 | TT | AA | breeding line |
| TX06A001386 | CC | GG | breeding line |
| MTS0721 | CC | GG | cultivar |
| NI04421 | TT | AA | cultivar |
| NE01481 | TT | AA | cultivar |
| NE06430 | TT | AA | cultivar |
| NE07688 | TT | AA | cultivar |
| NE08452 | TT | AA | cultivar |
| Scout 66 | CC | GG | cultivar |
| SD07W053 | CC | AA | cultivar |
| SD05085-1 | TT | AA | cultivar |
| SD06156-1 | CC | GG | cultivar |
| SD07056 | TT | AA | cultivar |
| SD07126 | TT | AA | cultivar |
| SD07165 | TT | AA | cultivar |
| SD08138 | TT | AA | cultivar |
| SD08145 | TT | GG | cultivar |
| SD08174 | TT | AA | cultivar |
| SD08198 | CC | GG | cultivar |
| Hitch | TT | AA | cultivar |
| Karl 92 | TT | AA | cultivar |
| Overland | CC | GG | cultivar |
| Ning7840/clark-NIL98 | TT | AA | breeding line |
| Ning7840/clark-09F-4 | TT | AA | breeding line |
| Ning7840/clark-09F-23 | TT | AA | breeding line |
| Ning7840/clark-09F-45 | TT | AA | breeding line |
| Ning7840/clark-NIL75 | TT | AA | breeding line |
| PHS2008F212bbb | TT | AA | breeding line |
| PHS2008F206bab | TT | AA | breeding line |
| Harry | CC | GG | cultivar |
| 2137 | TT | AA | cultivar |
| (W)Danby | CC | GG | cultivar |
| Armour | TT | AA | cultivar |
| ART | TT | AA | cultivar |
| Aspen | TT | AA | cultivar |
| Bill Brown | CC | GG | cultivar |
| CO050322 | CC | AA | cultivar |
| CO050337-2 | CC | GG | cultivar |
| Hatcher | CC | GG | cultivar |
| Hawken | TT | AA | cultivar |
| Ike | TT | AA | cultivar |
| Infinity CL | CC | GG | cultivar |
| JackPot | TT | AA | cultivar |
| Postrock | TT | AA | cultivar |
| Protection CL | TT | AA | cultivar |
| Santa Fe | TT | AA | cultivar |
| Shocker | TT | AA | cultivar |
| Smoky Hill | TT | AA | cultivar |
| T-136 | TT | AA | cultivar |
| T-140 | TT | AA | cultivar |
| T81 | CC | GG | cultivar |
| TAM 112 | CC | GG | cultivar |
| TAM111 | CC | GG | cultivar |
| TAM-110 | CC | GG | cultivar |
| Winterhawk | TT | AA | cultivar |
| Camelot | CC | GG | cultivar |
| Keota | TT | AA | cultivar |
| Alliance | CC | GG | cultivar |
| Lyman | TT | GG | cultivar |
| Wesley FHB1 | TT | AA | breedig line |
| Arapahoe | CC | GG | cultivar |
| Harding | CC | GG | cultivar |
| Rioblanco | TT | AA | cultivar |
| Heyne | TT | AA | cultivar |
| 2174 | TT | AA | cultivar |
| Jagalene | TT | AA | cultivar |
| Lakin (xianghui) | TT | AA | cultivar |
| TAM-304 | TT | AA | cultivar |
| 09-27-28 rec-989 | TT | AA | breeding line |
| 09-25-11 rec-124 | CC | GG | breeding line |
| 09-26-6 rec-679 | CC | GG | breeding line |
| 09S-100 277(FHB1)Trego F4 | CC | GG | breeding line |
| 09S-99 219(FHB1)Trego | TT | AA | breeding line |
| 09S-98 27(FHB1)Trego F4 | CC | GG | breeding line |
| 09S-103 262(FHB1)Wesley | TT | AA | breeding line |
| 09S-104 267(FHB1)Wesley F4 | TT | AA | breeding line |
| 09S-105 568(FHB1)Wesley F4 | TT | AA | breeding line |
| 09S-109 167(FHB1)Harding F4 | CC | GG | breeding line |
| 09S-108 61(FHB1)Harding F4 | CC | GG | breeding line |
| 09S-107 31(FHB1)Harding F4 | CC | GG | breeding line |
| Hondo | TT | AA | cultivar |
| TX06A001281 | CC | GG | breeding line |
| NW05M6015-25-4 | TT | AA | breeding line |
| T-151 | TT | GG | cultivar |
| KS970187-1-10 | TT | AA | breeding line |
| NX05M4180-6 | CC | GG | breeding line |
| T150-1 | TT | AA | breeding line |
| SD07184 | TT | AA | breeding line |
| KS08P1-108 | TT | AA | breeding line |
| Millennium | CC | GG | cultivar |
| AP04T8211 | CC | AA | breeding line |

Supplementary Table S3 Analysis of variance for SBWMV resistance in the RIL populations derived from crosses Deliver/OK03825-5403-6, Wesley/ OK03825-5403-6 and a natural population based on experiments conducted in Kansas State University, Manhattan KS, USA

| Populations |  | Degree of freedom | Sum of squared deviations | Mean square | F | P | h^2^ (%) |
| --- | --- | --- | --- | --- | --- | --- | --- |
| Deliver/ OK03825-5403-6 | Genotype | 262 | 2186.41 | 8.35 | 64.85 | 5.90E-295 | 96.50 |
|  | Environment | 1 | 0.00 | 0.00 | 0.03 | 0.8636 |  |
|  | GE interaction | 262 | 9.50 | 0.04 | 0.28 | 1 |  |
|  | Replication/Env | 2 | 2.57 | 1.29 | 10.00 | 5.46E-05 |  |
|  | Residuals | 524 | 67.43 | 0.13 |  |  |  |
| Wesley/ OK03825-5403-6 | Genotype | 177 | 1126.62 | 6.37 | 27.74 | 3.17E-139 | 84.90 |
|  | Environment | 1 | 6.12 | 6.12 | 26.66 | 4.05E-07 |  |
|  | GE interaction | 177 | 105.38 | 0.60 | 2.59 | 1.52E-14 |  |
|  | Replication/Env | 2 | 7.78 | 3.89 | 16.94 | 9.39E-08 |  |
|  | Residuals | 354 | 81.22 | 0.23 |  |  |  |
| Germplasms | Genotype | 158 | 934.99 | 5.92 | 55.04 | 2.14E-93 | 98.20 |
|  | Replication | 1 | 0.01 | 0.01 | 0.12 | 0.7328 |  |
|  | Residuals | 158 | 16.99 | 0.11 |  |  |  |

Supplementary Table S4 Identified SNP markers in *Sbwm1* region on *Ae. tauschii* physical map

| SNP_name | Genetic position on 5D (cM) | BAC_contig # |
| --- | --- | --- |
| GA8KES401AL4GG_122 | 184.178 | ctg5140 |
| GDS7LZN01CBWNE_99 | 184.269 | ctg5140 |
| GDS7LZN01C727R_132 | 184.269 | ctg5140 |
| Contig38521_41 | 184.269 | ctg5140 |
| Contig08039_464 | 184.36 | ctg5140 |
| GCE8AKX01AQCUQ_47 | 184.405 | ctg5140 |
| Contig08110_553 | 184.496 | ctg11101 |
| Contig29753_752 | 186.611 | ctg2530 |
| F1BEJMU02I00U2_83 | 186.656 | ctg2530 |
| GA8KES402HG9VM_327 | 187.432 | ctg2530 |
| GDS7LZN02I3554_251 | 187.706 | ctg2530 |
| GCE8AKX02IXEFJ_281 | 187.706 | ctg2530 |
| GDEEGVY01DSS5C_397 | 188.04 | ctg2530 |
| Contig21184_313 | 188.23 | ctg1806 |

Supplementary Table S5 Annotation of genes in the *Sbwm1* region

| Annotated in wheat | Annotated genes in *Ae. tauschii* | Gene function | Forward Primer | Reverse Primer |
| --- | --- | --- | --- | --- |
| TraesCS5D01G530300 | AET5Gv21176800 | RNA-binding protein Luc7-like 2 | - | - |
| TraesCS5D01G530400 | AET5Gv21176900 | Sister chromatid cohesion protein DCC1 | - | - |
| TraesCS5D01G530500 | —— | Poly (ADP-ribose) glycohydrolase activity | - | - |
| TraesCS5D01G530600 | AET5Gv21177000 | Protein kinase family protein, subfamily SD-2a | - | - |
| TraesCS5D01G530700 |  | Potassium channel activity (Blast2GO) | - | - |
| —— | AET5Gv21177600 | Conserved hypothetical protein | - | - |
| TraesCS5D01G530800 | AET5Gv21177700 | S-adenosylmethionine-dependent methyltransferase/ methyltransferase/ thiopurine | - | - |
| TraesCS5D01G530900 | AET5Gv21177800 | Poly (ADP-ribose) glycohydrolase activity | - | - |
| TraesCS5D01G531000 | AET5Gv21177900 | Pentatricopeptide repeat domain repeat family (PPR_2) // (PPR_3) | - | - |
| TraesCS5D01G531100 | AET5Gv21178000 | Zinc ion binding protein | - | - |
| TraesCS5D01G531200 | AET5Gv21178200 | Pto-interacting protein 1 (PTI1) | ACGACGACGACAGACATGAG | CGGTTTCCTTCCAGTCAGAA |
| TraesCS5D01G531300 | AET5Gv21178500 | Protein serine/threonine phosphatase activity | - | - |
| TraesCS5D01G531400 | AET5Gv21178600 | Zinc finger FYVE domain containing protein | - | - |
| TraesCS5D01G531500 | AET5Gv21178900 | Unknown function | - | - |
| TraesCS5D01G531600 | AET5Gv21179000 | Albino3-like protein 1 | - | - |
| TraesCS5D01G531700 | AET5Gv21179200 | Neutral amino acid transmembrane transporter activity, L-amino acid transmembrane transporter activity, aromatic amino acid transmembrane transporter activity | - | - |
| TraesCS5D01G531800 | AET5Gv21179300 | KDEL sequence binding, HDEL sequence binding | - | - |
| TraesCS5D01G531900 | AET5Gv21179700 | Protein of unknown function | - | - |
| TraesCS5D01G532000 | AET5Gv21179900 | Major pollen allergen-like protein | - | - |
| —— | AET5Gv21180000 | Domain of unknown function | - | - |
| TraesCS5D01G625100LC | AET5Gv21177400 | Zinc ion binding | - | - |
| TraesCS5D01G532100 | AET5Gv21180100 | RNA binding S1 | _ | _ |
